# Clinically Relevant Growth Conditions Alter *Acinetobacter baumannii* Antibiotic Susceptibility and Promote Identification of Novel Antibacterial Agents

**DOI:** 10.1371/journal.pone.0143033

**Published:** 2015-11-11

**Authors:** Jennifer M. Colquhoun, Rachel A. F. Wozniak, Paul M. Dunman

**Affiliations:** 1 Department of Microbiology and Immunology, University of Rochester School of Medicine and Dentistry, Rochester, New York, United States of America; 2 Department of Ophthalmology, Flaum Eye Institute, University of Rochester School of Medicine and Dentistry, Rochester, New York, United States of America; The Scripps Research Institute and Sorrento Therapeutics, Inc., UNITED STATES

## Abstract

Biological processes that govern bacterial proliferation and survival in the host-environment(s) are likely to be vastly different from those that are required for viability in nutrient-rich laboratory media. Consequently, growth-based antimicrobial screens performed in conditions modeling aspects of bacterial disease states have the potential to identify new classes of antimicrobials that would be missed by screens performed in conventional laboratory media. Accordingly, we performed screens of the Selleck library of 853 FDA approved drugs for agents that exhibit antimicrobial activity toward the Gram-negative bacterial pathogen *Acinetobacter baumannii* during growth in human serum, lung surfactant, and/or the organism in the biofilm state and compared those results to that of conventional laboratory medium. Results revealed that a total of 90 compounds representing 73 antibiotics and 17 agents that were developed for alternative therapeutic indications displayed antimicrobial properties toward the test strain in at least one screening condition. Of the active library antibiotics only four agents, rifampin, rifaximin, ciprofloxacin and tetracycline, exhibited antimicrobial activity toward the organism during all screening conditions, whereas the remainder were inactive in ≥ 1 condition; 56 antibiotics were inactive during serum growth, 25 and 38 were inactive toward lung surfactant grown and biofilm-associated cells, respectively, suggesting that subsets of antibiotics may outperform others in differing infection settings. Moreover, 9 antibiotics that are predominantly used for the treatment Gram-positive pathogens and 10 non-antibiotics lacked detectable antimicrobial activity toward *A*. *baumannii* grown in conventional medium but were active during ≥ 1 alternative growth condition(s). Such agents may represent promising anti-*Acinetobacter* agents that would have likely been overlooked by antimicrobial whole cell screening assays performed in traditional laboratory screening media.

## Introduction

Multi-drug resistant bacterial pathogens are responsible for significant morbidity, mortality and an estimated $20 billion annually in excess United States health care costs [1, 2]. The ESKAPE pathogens (*E* *nterococcus faecium*, *S* *taphylococcus auerus*, *K* *lebsiella pneumoniae*, *A* *cinetobacter baumannii*, *P* *seudomonas aeruginosa*, *and* *E* *nterobacter* sp.) are of particular concern as they not only “escape” current antibiotics but are also responsible for a significant portion of nosocomial infections [3]. As an example, a 2010 U.S. epidemiological survey revealed that 54% and 3.3% of *A*. *baumannii* clinical isolates tested were resistant to ≥ 3 and ≥ 6 classes of antibiotics, respectively [4]. Mortality rates associated with multidrug resistant *A*. *baumannii* infections are greater than 60% among susceptible patient populations [5] and are certain to increase due to the recent global emergence of extensively drug-resistant strains [6, 7]. In response, the Infectious Diseases Society of America and other agencies have called for renewed investment in the identification and development of novel antimicrobial agents for the treatment of bacterial pathogens, placing special emphasis on the ESKAPE pathogens [8].

Historically, the most successful means of antimicrobial identification has been whole-cell screening assays in which compound libraries are used to identify antimicrobial agents that limit an organism’s growth and/or viability in conventional laboratory medium. However, these screening campaigns repeatedly identify the same classes of antimicrobial agents for which resistance determinants are already circulating [9, 10]. In response, Silver and colleagues have advocated two approaches to circumvent this issue. The first is to expand small molecule library chemical space in order to identify structurally novel classes of antibiotics. The second is to implement novel screening methods that allow “seeing what had previously not been seen” within existing compound libraries [9].

With regard to the latter, it is clear that growth in conventional laboratory media does not accurately represent the conditions that bacterial pathogens encounter during host colonization, pathogenesis and treatment conditions [11–13]. Indeed, we and others have shown that the cellular processes that govern bacterial growth and survival in nutrient rich laboratory media are likely to be vastly different from those that are required for growth in media that more accurately recapitulates aspects of the host environment(s) [14, 15]. As such, cellular processes that are nonessential for growth in conventional laboratory medium may take on more importance and be required for adaptation, proliferation, and/or survival within the host. Corresponding inhibitors of these processes/targets are likely to represent novel antimicrobials that are missed by whole cell growth based assays performed in conventional laboratory media. Recognizing this, Fahnoe and colleagues recently identified antimicrobial compounds that inhibit the *P*. *aeruginosa* glyoxylate shunt pathway, which is required for the organism’s viability in the lungs, by screening an existing chemical library for growth inhibition in nutrient-restrictive media that more closely represents this *in vivo* environment [11]. Results of that screening campaign identified glyoxylate shunt inhibitors that displayed excellent antimicrobial properties in nutrient-limited media but did not affect *P*. *aeruginosa* grown in conventional laboratory media. While it remains to be seen whether the glyoxylate shunt inhibitors identified in that study will display clinical efficacy, their data indicate that novel classes of antimicrobials can be identified in whole cell assays of existing chemical libraries that are performed in growth states/conditions resembling the host environment.

Whole cell antimicrobial screening in biologically relevant growth conditions also allows for the potential to overcome host condition-associated adaptive changes in bacterial physiology as well as unforeseen host-drug interactions that could limit the effectiveness of antimicrobial compounds *in vivo*. For instance, during growth in physiological salt conditions or human serum *A*. *baumannii* induces adaptive efflux-mediated antibiotic resistance mechanisms that limit the effectiveness of current antibiotics [14, 16]. Consequently, screening for agents with antimicrobial activity toward *A*. *baumannii* grown in either low salt or human serum would presumably allow for the identification of antimicrobial compounds that overcome adaptive antibiotic resistance. Similarly, antimicrobial programs focused on the identification or improving an agent’s performance in conventional media cannot foresee complications of drug and host-factor interactions that limit antibiotic efficacy, such as the inactivation of daptomycin by lung surfactant lipid aggregates [17].

Accordingly, the goal of this current work was to interrogate the Selleck library of Food and Drug Administration (FDA) approved drugs for agents that display antimicrobial activity toward *A*. *baumannii* during growth in conventional laboratory medium, human serum, lung surfactant and/or biofilm state, as a means to formally evaluate whether novel agents can be identified by screening in these alternative conditions. Minimum inhibitory concentration (MIC) testing of eight classes of antibiotics that are present within the Selleck library was initially performed to establish the strain’s susceptibility profile and, consequently, to serve as controls to ensure library screening was performing as expected. Interestingly, MIC testing revealed that *A*. *baumannii* displays pronounced variability in susceptibility to several antibiotics when grown in alternative growth conditions. Expanded testing of the ESKAPE pathogens *S*. *aureus*, *K*. *pneumoniae* and *P*. *aeruginosa* displayed similar differences, suggesting that certain classes of antibiotics may be more valuable than others in specific infection settings. Further, Selleck library screens identified 19 compounds that exhibit antimicrobial activity toward *A*. *baumannii* in the above mentioned clinically relevant media that displayed no activity toward the organism grown in conventional laboratory medium. These agents may represent progenitor scaffolds for new classes of anti-*Acinetobacter* agents.

## Materials and Methods

### Bacterial strains and growth conditions

The bacterial strains used in this study are listed in Table 1. Unless otherwise indicated, bacteria were grown for 16 hours in Mueller-Hinton (MH) (Becton, Dickinson, Franklin Lakes, NJ) medium at 37°C on a rotary shaker at 225 rotations per minute (rpm) and then used to inoculate (1:100) fresh MH, fresh human serum (MP Biomedicals, Solon, OH) or bovine pulmonary surfactant (Infasurf, ONY, Amherst, NY) medium and processed, as described below.

**Table 1:** Bacterial strains used in this study.

Species	Strain	Reference
*Staphylococcus aureus*	UAMS-1	[37]
*Pseudomonas aeruginosa*	PA01	[38]
*Klebsiella pneumoniae*	cKP4	[39]
*Acinetobacter baumannii*	98-37-09	[20]

### Chemicals

The Selleck Chemical Library of 853 FDA-approved drugs was acquired from Selleck Chemical (Houston, TX). ToxiLight BioAssay kits were obtained from Lonza (Basel, Switzerland). Sulfamethoxazole, erythromycin, ciprofloxacin, ampicillin and minocycline were purchased from Sigma-Aldrich (St. Louis, MO). Linezolid and vancomycin were purchased from Thermo Fisher (Waltham, MA). Daptomycin was purchased from Biotang Inc (Lexington, MA).

### Compound library screening of *A*. *baumannii* grown in Mueller-Hinton (MH), serum, or lung surfactant medium

Members of the Selleck compound library were screened for antimicrobial activity toward *A*. *baumannii* during growth in MH, serum, or lung surfactant. To do so, an overnight culture of *A*. *baumannii* strain 98-37-09 was used to inoculate (1:100 dilution) 25 ml fresh MH medium, 100% serum or 100% lung surfactant and grown to exponential phase at 37°C in a rotary shaker at 225 rpm. Cultures were then diluted to a cell density of ~3 x 10^7^ colony forming units (CFU) ml^-1^ in the same medium as used for propagation. Ten microliters of diluted culture (~3 x 10^5^ CFU per well) was added to individual wells of a 96-well round-bottom polystyrene plate containing 89 μl of the same medium as used for propagation and 1 μl of a 5 mmol antibiotic stock solution from the Selleck Chemical library (50 μmol final concentration). DMSO was used as a negative control since stock chemicals were prepared in DMSO. Well constituents were mixed by pipetting and plates were incubated at 37°C for 16 hours. Compounds scored as exhibiting antimicrobial activity toward MH or serum grown bacteria were identified by the lack of visible growth after incubation. Plates containing surfactant were opaque making scoring for bacterial growth difficult by eye; thus well constituents were diluted and plated (10^−5^ final dilution) to identify compounds that elicited a 2-log CFU ml^-1^ decrease in cell growth inhibition in comparison to vehicle (DMSO) treated cells. All compounds which resulted in decreased cell growth were confirmed by repeat testing in duplicate.

### Compound library screening of established *A*. *baumannii* biofilms

Members of the Selleck library were screened for compounds that displayed bactericidal activity toward 24 h established *A*. *baumannii* biofilms using the adenylate kinase (AK) cell-death reporter assay, as previously described [18]. Briefly, *A*. *baumannii* strain 98-37-09 was cultured overnight in MH medium and then used to seed 96-well, flat-bottom polystyrene plates. Plates were incubated at 37°C in a humidified incubator for 24 h to allow static biofilm formation. Non-adherent cells were removed by aspiration and biofilms were washed twice with sterile phosphate-buffered saline (PBS). Fresh MH medium supplemented with 50 μM of test compound was added to each well and incubated overnight at 37°C. Following treatment, 100 μl of supernatant was transferred to 96-well, white-walled plates, 100 μl of ToxiLight AK reagent was added to each well and mixtures were incubated for 30 min at room temperature. Luminescence was measured using a SpectraMax M5 plate reader. An antimicrobial hit was defined as a compound that elicited a 2-fold increase in AK signal as compared to the vehicle (DMSO) treated cells. Each hit was reconfirmed by repeat testing in duplicate.

### Minimal inhibitory concentration (MIC) testing

MIC testing was performed for the indicated antibiotics and test compounds. For antimicrobial testing of bacteria grown in MH or serum, 10 μl of an overnight culture (~3 x 10^5^ CFU) were added to individual wells of a 96-well round-bottom plate containing 88 μl of MH or serum and 2 μl of test compound (ranging from 0 to 100 μM) or known antibiotics (ranging from 0 to 256 μg ml^-1^). Mixtures were incubated at 37°C for 16 hours and the MIC was defined as the lowest concentration of compound in which no visible cell growth was observed by the unaided eye. The minimum inhibitory concentration of compounds toward bacteria grown in lung surfactant was determined as the lowest concentration of the test agent that reduced bacterial growth ≥ 2-log CFU ml^-1^ in comparison to mock treated cells. To determine the MIC of test compounds against biofilms, *A*. *baumannii* static biofilms were grown and washed, as described above, and then treated with fresh MH medium supplemented with 2 μl of an antibiotic solution (ranging 0 to 256 μg ml^-1^) or test compound (ranging from 0 to 100 μM) then incubated overnight at 37°C. Following treatment, biofilm-associated cells were washed, disrupted by pipetting and vortexing vigorously then plated at 10^−5^ dilution to determine the lowest antibiotic or test compound concentration that elicited a 2-log CFU ml^-1^ decrease in comparison to vehicle (DMSO) treated biofilms.

## Results

### Growth conditions affect the antimicrobial activity of antibiotics toward *A*. *baumannii*

The Selleck library, which is comprised of 853 FDA approved drugs (including 88 antibiotics), was screened for agents that displayed antimicrobial activity toward *A*. *baumannii* grown in Mueller-Hinton (MH) media, human serum, lung surfactant or biofilms and compared to determine whether compound screening in alternative bacterial growth media would allow for the identification of novel antimicrobial agents that are missed by conventional screens in nutrient-rich media. As a prerequisite to doing so, the antimicrobial susceptibility profile of the test *A*. *baumannii* strain 98-37-09, a well-characterized clinical isolate that forms robust biofilms [19] and grows well in both human serum [20] and lung surfactant (unpublished), to eight classes of antibiotics represented within the library was determined as a means to establish their performance expectations during high throughput screening conditions.

As shown in Table 2, MIC testing in conventional MH media revealed that the strain was susceptible to ampicillin (8 μg ml^-1^), ciprofloxacin (0.5 μg ml^-1^), erythromycin (16 μg ml^-1^) sulfamethoxazole (2 μg ml^-1^), and minocycline (0.5 μg ml^-1^). As expected, daptomycin, vancomycin, and linezolid, which are used for the treatment of Gram-positive pathogens, where not efficacious displaying MIC values of > 256 μg ml^-1^, 256 μg ml^-1^, and 128 μg ml^-1^, respectively. Interestingly, during serum growth the test strain exhibited increased antimicrobial susceptibility toward vancomycin (16-fold; 16 μg ml^-1^), erythromycin (32-fold; 0.5 μg ml^-1^), and sulfamethoxazole (4-fold; 0.5 μg ml^-1^). Likewise, during growth in lung surfactant the strain exhibited significant increases in susceptibility to ampicillin (16-fold; 0.5 μg ml^-1^), vancomycin (32-fold; 8 μg ml^-1^), sulfamethoxazole (4-fold; 0.5 μg ml^-1^) and linezolid (4-fold; 32 μg ml^-1^).

**Table 2:** Antimicrobial susceptibility measures.

	Minimum Inhibitory Concentration (μg ml^-1^)*							
Species (strain)	Amp	Dap	Cipro	Van	Erm	Smz	Linz	Mino
***A*. *baumannii* (98-37-09)**								
MH	8	>256	0.5	256	16	2	128	0.5
Serum	8	>256	0.5	16	0.5	0.5	128	0.5
Surfactant	0.5	>256	2	8	16	0.5	32	0.5
Biofilm	>256	>256	>256	>256	>256	>256	>256	>256
***P*. *aeruginosa* (PA01)**								
MH	256	>256	0.5	>256	64	256	>256	16
Serum	64	>256	0.5	32	8	32	128	>256
Surfactant	256	>256	4	>256	256	64	>256	32
***K*. *pneumoniae* (cKP4)**								
MH	64	>256	256	>256	128	256	256	2
50% Serum	16	>256	0.5	256	8	2	256	8
Surfactant	64	>256	0.5	>256	>256	8	>256	4
***S*. *aureus* (UAMS-1)**								
MH	1	1	0.5	1	0.5	1	1	0.5
Serum	2	16	0.5	2	0.5	128	2	1
Surfactant	0.5	>256	1	1	4	16	1	0.5

* Ampicillin (Amp); Daptomycin (Dap); Ciprofloxacin (Cipro); Vancomycin (Van); Erythromycin (Erm); Sulfamethoxazole (Smz); Linezolid (Linz); Minocycline (Mino).

The observed increase in susceptibility of *A*. *baumannii* to a subset of antibiotics tested during growth in human serum and/or lung surfactant may reflect corresponding changes in the organism’s physiology during growth in alternative media and may have expanded clinical significance by indicating how these agents might perform in various infection settings. To determine whether this phenomenon was *A*. *baumannii* specific, studies were expanded to include three additional members of the ESKAPE pathogens, *P*. *aeruginosa*, *K*. *pneumoniae*, and *S*. *aureus*.

### Growth conditions affect the antimicrobial activity of antibiotics toward *P*. *aeruginosa*, *K*. *pneumoniae and S*. *aureus*

As shown in Table 2, MIC testing revealed that while *P*. *aeruginosa* strain PA01 displayed similar susceptibility profiles for most antibiotics during growth in both MH and lung surfactant, the strain exhibited increased susceptibility toward ampicillin (4-fold; 64 μg ml^-1^), vancomycin (6-fold; 32 μg ml^-1^), erythromycin (6-fold; 32 μg ml^-1^), and sulfamethoxazole (8-fold; 32 μg ml^-1^) during serum growth. Conversely, PA01 displayed a pronounced increase in tolerance to the antibiotic minocycline (8-fold; > 256 μg ml^-1^) in serum, as previously described [21].

The *K*. *pneumoniae* strain evaluated, cKP4, was resistant to daptomycin, ciprofloxacin, vancomycin, erythromycin, sulfamethoxazole and linezolid during growth in MH medium (MICs ≥ 128 μg ml^-1^). Despite this, the strain displayed increased susceptibility to ciprofloxacin (512-fold; 0.5 μg ml^-1^) and sulfamethoxazole (2 and 8 μg ml^-1^) when grown in serum or lung surfactant, respectively (Table 2).

Minimum inhibitory concentration testing revealed that *S*. *aureus* strain UAMS-1 was susceptible to each of the antibiotics tested (MICs ≤ 1.0 μg ml^-1^) during growth in conventional MH medium. During growth in both serum and surfactant the strain showed similar susceptibility profiles for the antibiotics ampicillin, ciprofloxacin, vancomycin, linezolid, and minocycline. Conversely, the organism exhibited decreased susceptibility to the antibiotics daptomycin, erythromycin and sulfamethoxazole during growth in serum and/or lung surfactant. More specifically, the MIC of daptomycin increased 16-fold and 256-fold during *S*. *aureus* growth in serum and surfactant, respectively. These results support those of Silverman and colleagues who found surfactant lipid aggregates inhibit daptomycin’s antibacterial activity and likely contribute to the agent’s limited therapeutic efficacy in treating patients with community acquired pneumonia [17]. Sulfamethoxazole displayed 128-fold and 16-fold decreases in activity toward serum and surfactant grown *S*. *aureus*, respectively.

Taken together, these results indicate that growth conditions directly or indirectly affect the potency of subsets of antibiotics and, by extension, that their efficacy may be dictated, in part, by an invading pathogen’s infection setting. Of direct relevance to the current study, these results also provide circumstantial evidence that bacterial physiology is likely to be vastly different in growth in alternative media, providing opportunity to identify agents that target cellular processes that may not be essential for growth in nutrient rich conditions, but required for growth in alternative growth conditions. To test this hypothesis, we screened the Selleck compound library of FDA-approved drugs toward *A*. *baumannii* during growth in conventional laboratory medium (MH), human serum, pulmonary surfactant, and established biofilms.

### Selleck Compound Library Screening

*A*. *baumannii* Selleck library screens (50 μM) were performed in MH medium, 100% human serum, 100% bovine lung surfactant or 48-hr established biofilm associated cells. The antimicrobial effects of test compounds toward the organism in MH and serum were measured visually as growth inhibition, whereas activity in surfactant was determined by dilution plating. Biofilm associated bacterial killing was monitored by adenylate kinase release, as previously described [18]. A total of 90 drugs displayed antimicrobial activity toward *A*. *baumannii* strain 98-37-09 during ≥ 1 of the growth conditions which was confirmed with repeat testing; known antibiotics are listed in Table 3 whereas drugs developed for alternative therapies are listed in Table 4.

**Table 3:** Selleck library antibiotic activities.

	Selleck Screening Results^1^				Minimum Inhibitory Concentration^2^			
Compound	MH	Serum	Surfactant	Biofilm	MH	Serum	Surfactant	Biofilm
Doripenem	X	X	X					
Meropenem	X	X	X					
Tigecycline	X	X	X					
Rifampin	X	X	X	X				
Rifaximin	X	X	X	X				
Ciprofloxacin	X	X	X	X	<0.39	1.56	6.25	1.8
Tetracycline	X	X	X	X				
Bleomycin sulfate	X		X	X				
Gatifloxacin	X		X					
Biapenem	X		X	X				
Ofloxacin	X		X	X				
Marbofloxacin	X		X	X				
Moxifloxacin	X		X	X				
Cefdinir	X		X					
Rifapentine	X		X	X				
Rifabutin	X		X	X				
Pefloxacin	X		X	X				
Sparfloxacin	X		X	X				
Sulbactam	X		X	X				
Sarafloxacin	X		X	X				
Balofloxacin	X		X	X				
Tebipenem	X		X					
Fleroxacin	X		X	X				
Sitafloxacin	X		X	X				
Methacycline	X		X	X	<0.39	>100	<12.5	1.2
Besifloxacin	X		X	X	0.78	<12.5	50	1.7
Enrofloxacin	X		X	X				
Flumequine	X		X	X				
Nadifloxacin	X		X	X	0.78	>100	<12.5	2.9
Clinafloxacin	X		X	X				
Azlocillin	X		X					
Levofloxacin	X	X		X				
Trimethoprim	X	X						
Ampicillin	X	X	X					
Nalidixic acid	X	X	X					
Doxycycline		X	X	X				
Norfloxacin	X							
Erythromycin	X			X				
Azithromycin	X			X				
Chloroxine	X			X				
Lomefloxacin	X			X				
Clarithromycin	X			X				
Streptomycin	X			X				
Danofloxacin	X			X				
Pyrithione	X			X				
Cefoperazone	X		X					
Aztreonam	X		X					
Sulfamethoxazole	X		X					
Sulfapyrdine	X		X					
Tobramycin	X		X					
Sulfathiazole	X		X					
Sulfameter	X		X					
Novobiocin	X	X						
Amoxicillin	X	X						
Pentamidine	X	X			100	50	>100	NC
Amikacin	X	X						
Vancomycin			X	X	>100	100	<12.5	1.4
Sulfisoxazole	X							
Neomycin sulfate	X							
Roxithromycin	X							
Sulfamerazine	X							
Netilmicin sulfate	X							
Sulfadiazine	X							
Sulfamethizole	X							
Sulfanilamide	X							
Dequalinium chloride	X				50	100	>100	NC
Clindamycin		X						
Cefaclor			X					
Cephalexin			X					
Oxytetracycline			X					
Nitrofurazone			X					
Cefprozil			X					
Oxacillin			X					

^1^ X indicates growth inhibitory phenotype in the indicated screening condition, measured as no visible growth in Mueller-Hinton (MH) or serum; ≥ 2-log decrease in colony forming units in lung surfactant; ≥ 2-fold increase in adenylate kinase signal in comparison to DMSO treated biofilms.

^2^ Measures in μM; No change detected (NC); biofilm values presented as log_10_ decrease.

**Table 4:** Selleck library non-antibiotic activities.

	Selleck Screening Results^1^				Minimum Inhibitory Concentration^2^			
Compound	MH	Serum	Surfactant	Biofilm	MH	Serum	Surfactant	Biofilm
Epirubicin hydrochloride	X				100	>100	>100	NC
Idarubicin HCl	X				25	>100	>100	NC
Nebivolol (Bystolic)	X				100	>100	>100	NC
Pyrimethamine	X				50	>100	>100	NC
Zinc pyrithione	X			X	25	> 100	> 100	25
Ciclopirox	X		X		50	>100	25	NC
Ribavirin	X		X		100	50	12.5	NC
Zoledronic acid			X		>100	>100	25	NC
Ronidazole			X		>100	50	25	NC
Telaprevir			X		>100	>100	25	NC
Cisatracurium besylate			X		>100	>100	100	NC
Cisplatin			X		>100	>100	6.25	NC
Hydralazine hydrochloride			X		>100	>100	50	NC
Nitazoxanide			X		>100	>100	12.5	NC
Fluorouracil			X		>100	100	0.78	NC
Azasetron HCl			X		>100	>100	100	NC
Doxorubicin		X			>100	50	>100	NC

^1^ X indicates growth inhibitory phenotype in the indicated screening condition, measured as no visible growth in Mueller-Hinton (MH) or serum; ≥ 2-log decrease in colony forming units in lung surfactant; ≥ 2-fold increase in adenylate kinase signal in comparison to DMSO treated biofilms.

^2^ Measures in μM; No change detected (NC); biofilm values presented as log_10_ decrease.

A survey of screening results for each control antibiotic’s performance closely resembled each agent’s activity in the aforementioned pilot susceptibility studies, indicating that Selleck library screening conditions were appropriate for measuring antimicrobial activities of test compounds (Table 3). Indeed, as expected, screening results revealed that the strain displayed: 1. Daptomycin and linezolid resistance during all test conditions (not shown), 2. Susceptibility to ampicillin and ciprofloxacin during growth in MH, serum, and lung surfactant, 3. Susceptibility to erythromycin, sulfamethoxazole, and minocycline during growth in MH, and 4. Increased susceptibility toward vancomycin and sulfamethoxazole in alternative growth media. More specifically, while vancomycin did not exhibit a measurable antimicrobial effect in MH media, the antibiotic did exhibit antibacterial activity during growth in lung surfactant, whereas sulfamethoxazole displayed antimicrobial activity toward the test strain in lung surfactant and in MH. However, we did also note differences between the performance of individual antibiotics in initial testing and the high throughput setting. Both vancomycin and ciprofloxacin exhibited antimicrobial activity toward biofilm associated cells in the high throughput screen (but not during initial test conditions; Table 2). Repeat testing in which biofilm associated cells were treated with drug and then plated to enumerate cell viability revealed that ciprofloxacin and vancomycin treatment resulted in a 1.8-log and 1.4-log reduction in biofilm-associated *A*. *baumannii*, respectively, providing confidence that the screen performed adequately (**Table 3**). Nonetheless, erythromycin and sulfamethoxazole, both of which exhibited activity toward serum and surfactant grown cells in initial tests (Table 2), failed to display activity in these medium conditions in the Selleck library screen for reasons unknown to us, indicating that the high throughput assays performed do have limitations.

An overall assessment of screening results revealed that the majority (73 of 90; 81%) of drugs exhibiting antimicrobial activity in at least one growth condition represented known antibiotics, which are listed in Table 3. Of these, only four antibiotics (5.4%), rifampin, rifaximin, ciprofloxacin and tetracycline, exhibited antibacterial activity during all growth states, whereas most (95.6%) lacked activity in at least one condition. More specifically, 9 antibiotics (12.3%) exhibited antimicrobial activity solely in MH. Twenty four antibiotics (32.8%) were active toward the test strain in MH and one other screening condition (5 serum; 11 surfactant; 8 biofilms), whereas 26 antibiotics were active toward the organism in MH and two of the three other screening conditions evaluated (5 serum and surfactant; 20 surfactant and biofilms; 1 serum and biofilm). Nine antibiotics (12.3%), including vancomycin, clindamycin, cefaclor, cephalexin and oxacillin, which are predominantly used for the treatment of Gram-positive bacterial infections did not display antimicrobial activity in MH, but did exhibit activity in at least one alternative growth condition. Standard antimicrobial susceptibility testing of five randomly selected antibiotics, methacycline, besifoxacin, nadifloxacin, pentamidine, and dequalinium, confirmed our screening results (Table 3). While the antibiotic colistin was not a member of the Selleck library screened it is often used as a last-line therapy for *A*. *baumannii* infection. Thus, standard antimicrobial testing was also performed for colistin in each media/growth condition. Results revealed that during growth in Mueller-Hinton medium *A*. *baumannii* strain 98-37-09 displayed an MIC of 2 μg ml^-1^ toward colistin, increased susceptibility during growth in human serum (0.12 μg ml^-1^), but little or no activity toward the organism when grown in lung surfactant or in the biofilm state (> 256 μg ml^-1^ and 128 μg ml^-1^, respectively).

Selleck library screening and conventional antimicrobial assays subsequently confirmed that a total of 17 non-antibiotic drugs that have been developed for alternative therapeutic indications also displayed antimicrobial activity toward *A*. *baumannii* in ≥ 1 test condition, suggesting that they have the potential to be repurposed as antibacterial agents (Table 4). More specifically, four drugs, epirubicin, idarubicin, nebivolol and pyrimethamine, exhibited antibacterial activity toward the test strain during growth in MH but no other screening condition. The antiseptic, zinc pyrithione, exhibited activity toward cells in MH and biofilm-associated cells. Two compounds, ciclopirox and ribavirin, demonstrated activity in MH and lung surfactant. Eight drugs only displayed activity toward surfactant grown cells, whereas, doxorubicin, only showed activity toward serum grown cells but no other test conditions.

Taken together, our results support the hypothesis that bacteria are likely to rely on different cellular processes during growth in biologically relevant conditions that are not required for survival in conventional laboratory medium. Corresponding differences in the physiology of bacterial cells appear to affect the organism’s susceptibility to current (and future) antibiotics, offer opportunity to exploit new targets for antimicrobial development and allow the identification of new antibacterials within existing well-characterized compound libraries.

## Discussion

Most bacterial pathogens will encounter a variety of growth conditions both within its host and the environment. As such, organisms require several, and often distinct, cellular processes to endure and propagate under these different growth conditions. Recent reports have highlighted the importance of specific growth states of bacterial pathogens as it relates to antibiotic susceptibility. This is perhaps best exemplified by the clinical failure of antibiotics to effectively treat otherwise antibiotic susceptible bacteria within established biofilms or small colony variant growth state (reviewed in [22, 23]).

Accordingly, it follows that bacteria are likely to rely on different cellular processes during growth in biologically relevant growth media that are not required for survival in conventional laboratory medium. Corresponding differences in the physiology of bacterial cells may affect the organism’s susceptibility to current (and future) antibiotics and offer the opportunity to exploit new targets for antimicrobial development. Indeed, a recent study by Lee *et al*., identified *P*. *aeruginosa genes* that were essential for propagation in a clinically relevant cystic fibrosis sputum media as compared to standard nutrient-rich media (Luria Broth) and/or minimal media [24]. The authors found that functions associated with outer membrane synthesis and integrity to be essential only in sputum media, thereby illustrating how bacterial physiology is altered dependent on environmental conditions and provide further insight into a pathogen’s pathophysiology. Thus, pathways that remain “hidden” when the organism is grown in nutrient-rich media may become viable antibacterial targets during alternative growth conditions.

As antibiotic resistance becomes an increasingly pressing health care concern, it is necessary not only to search for novel compounds but to broaden our investigation into existing FDA-approved compounds for efficacy against pathogens in growth conditions that more closely resemble clinically relevant states. By focusing on serum, pulmonary surfactant, and biofilms, we aimed to more closely mimic the environment that *A*. *baumannii* as well as other ESKAPE pathogens encounter during infection within the bloodstream, lung, and abiotic surfaces such as orthopedic transplants or catheters, respectively. These infection sites are important clinically with bloodstream infections comprising 40%, pneumonia 10%, and orthopedic transplants and catheters 23% of hospital acquired infections [25].

The antimicrobial susceptibility profile of 8 classes of antibiotics toward the *A*. *baumannii* test strain, 98-37-09, revealed substantial growth condition-specific differences in the potency of these compounds. In fact, several antibiotics displayed improved anti-*Acinetobacter* performance during serum or surfactant growth which may reflect increased cellular dependence on antibiotic targeted pathways, onset of antibiotic “off-target” alternative activities, or reduction in intrinsic antibiotic tolerance mechanisms. Most notably we observed that despite the fact that the test strain demonstrated resistance to the Gram-positive antibiotic, vancomycin, during growth in Mueller-Hinton media, it demonstrated susceptibility during growth in lung surfactant and biofilms. This uncovered susceptibility to vancomycin in a clinically relevant media could indicate potential efficacy in the treatment of *A*. *baumannii* associated pneumonia and represent an additional antibiotic to target this organism that would not have previously been considered. Indeed, others have similarly observed that while the *A*. *baumannii* strain ATCC 19606 is resistant to vancomycin during growth in MH media, the antibiotic exhibits efficacy toward the strain in an invertebrate model of *A*. *baumannii* infection [26]. Additionally, we observed that growth of the test strain in lung surfactant improved its susceptibility to ampicillin. However, expanded testing of a panel of 13 genetically distinct *A*. *baumannii* clinical isolates [20] revealed that although all strain’s tested did indeed display improved ampicillin activity during growth in lung surfactant, the extent of improvement was more modest and varied per test strain (data not shown).

We also observed that other representatives of the ESKAPE pathogens, *P*. *aeruginosa*, *K*. *pneumoniae*, and *S*. *aureus*, also displayed varied responses to antibiotics dependent on the growth media. The decreased efficacy of daptomycin and sulfamethoxazole against *S*. *aureus* in serum and surfactant warrants consideration when considering clinical practice patterns in the treatment of *S*. *aureus* pneumonia and bloodstream infections. Increased susceptibility to ampicillin, vancomycin, erythromycin, sulfamethoxaxole in *P*. *aeruginosa* and ciprofloxacin and sulfamethoxazole against *K*. *pneumoniae* may all represent broadened applications for these existing FDA-approved antibiotics.

Selleck library screening also revealed 17 non-antibiotic drugs that have been developed for alternative therapeutic indications displayed antimicrobial activity against *A*. *baumannii* when the organism is grown in one or more clinically relevant conditions, suggesting that they have the potential to be repurposed as antibacterial agents (Table 4). Although considered non-antibiotics epirubicin [27], idarubicin [28], pyrimethamine [29], ciclopirox [30], ribavirin [31], ronidazole [32], cisplatin [33], nitazoxanide [34], fluorouracil [35], and doxorubicin [36] have been previously shown to exhibit antibacterial properties toward at least one bacterial species. Conversely, zoledronic acid, cisatracurium besylate, telaprevir, azasetron, which were all identified to exhibit anti-*Acinetobacter* activity toward surfactant grown cells (but not during nutrient rich conditions) have not been previously described to display antimicrobial properties to our knowledge and may represent new classes of antimicrobial agents. Currently our laboratory is actively investigating the underlying mechanisms that may modulate the observed changes in antibiotic susceptibility in response to growth in these alternative media conditions. Aside from the obviously inherit differences in nutrition sources and bacterial physiology, we have considered that the pH of differing media conditions may account for susceptibility changes that we have reported here. Yet, pH measures of Mueller-Hinton, serum, and of biofilm medium all are very similar both prior to- and following- *A*. *baumannii* growth (7.76 ± 0.33 and 8.29 ± 0.39, respectively), suggesting that differing media pHs are unlikely to contribute to corresponding alterations in bacterial antibiotic susceptibility between these three growth conditions. Conversely, pH may, in part, contribute to alterations in bacterial susceptibility to growth in lung surfactant, which has a pH of 4.73 but raises to 8.03 following bacterial incubation.

Taken together, our results demonstrate the importance of considering growth conditions in the investigation of novel antibacterial agents and, if subsequently clinically substantiated, an area that of opportunity for expanded CLSI guideline testing. Pathogens can often occupy several niches in a host each associated with both up- and down-regulation of specific biochemical pathways. Thus it is critical to develop new antibiotics with these varied growth states in mind as this will likely provide the greatest efficacy in treating human disease. Additionally, utilizing FDA-approved compound library screens allows for the identification of existing therapeutics that can be quickly repurposed and may be better tolerated in future animal studies.
